# Behavioral Changes in *Caenorhabditis elegans* After Exposure to Radial Extracorporeal Shock Waves

**DOI:** 10.3390/jcm14207206

**Published:** 2025-10-13

**Authors:** Tanja Hochstrasser, Leon Kaub, Leonard Maier, Nicholas B. Angstman, Tomonori Kenmoku, Carmen Nussbaum-Krammer, Christoph Schmitz

**Affiliations:** 1 Department of Anatomy II, Ludwig-Maximilians-University of Munich, 81366 Munich, Germany; leon.kaub@med.uni-muenchen.de (L.K.); lpn.maier@gmail.com (L.M.); carmen.nussbaum@med.uni-muenchen.de (C.N.-K.); christoph_schmitz@med.uni-muenchen.de (C.S.); 2 Department of Orthopedic Surgery, Kitasato University School of Medicine, Sagamihara 252-0329, Japan; kenmoku@med.kitasato-u.ac.jp

**Keywords:** *Caenorhabditis elegans*, nAChR, rESWs, nicotine, carbachol, cerebral palsy, spasticity

## Abstract

**Background/Objectives**: Cerebral palsy (CP) is a leading cause of motor disability in children and is commonly associated with spasticity. Treatment with radial extracorporeal shock waves (rESWs) is an established non-invasive therapy for spasticity, although its underlying mechanisms remain poorly understood. *Caenorhabditis elegans* (*C. elegans*) represents a powerful model for neuromuscular research due to its fully mapped nervous system, conserved cholinergic pathways and suitability for high-throughput behavioral analysis. This study aimed to test whether rESWs modulate cholinergic signaling at the neuromuscular junction (NMJ) in *C. elegans*. **Methods**: Wild-type and *acr-16* mutant *C. elegans* were exposed in liquid to varying doses of rESWs, nicotine and carbachol in different combinations. Locomotor behavior was recorded using high-resolution video tracking, and parameters including peristaltic speed, body wavelength, reversals and omega bends were quantified. **Results**: Exposure to rESWs transiently altered locomotion, most notably by reducing forward speed and increasing the frequency of reversals. However, rESWs did not consistently modify behavioral responses to nicotine or carbachol, and these effects were not clearly dependent on NMJ-associated nicotinic receptors. **Conclusions**: Exploring *C. elegans* as a model for rESW effects on spasticity proved informative but also revealed important limitations. Results indicate that rESWs act on the nervous system more broadly, extending beyond neuromuscular structures. This contrasts with the clinical situation, where rESWs primarily target muscles and connective tissues. While this precludes *C. elegans* as a direct model for CP-related spasticity, the observation that rESWs influence nervous-system function at a systemic level points to potential therapeutic avenues for neurological diseases.

## 1. Introduction

Cerebral palsy (CP) is a leading cause of motor disability in children and is commonly associated with spasticity, a condition characterized by increased muscle tone and exaggerated reflexes [1]. Managing spasticity is critical to improving mobility and quality of life in individuals with CP [1].

Among the available treatment options, radial extracorporeal shock wave therapy (rESWT) has emerged as an established non-invasive modality for reducing spasticity due to CP [2,3,4]. Radial extracorporeal shock waves (rESWs) are applied transcutaneously and penetrate tissues to a depth of up to 2 cm [5]. Clinical studies have demonstrated that rESWT can reduce spasticity and improve motor function both immediately and for up to three months after repeated treatments [6]. Despite its therapeutic potential, the molecular and cellular mechanisms underlying these beneficial effects remain largely unknown.

To explore the mechanisms of rESWT, we have previously shown that in intact rat muscles, rESWs induce a reduction and degeneration of acetylcholine receptors (AChRs), along with a selective breakdown of the neuromuscular junction (NMJ). This results in a transient disruption of neuromuscular transmission and a measurable decrease in compound muscle action potentials [7,8,9].

Acetylcholine receptors (AChRs) are activated by the neurotransmitter acetylcholine (ACh) and are expressed in both the central nervous system (CNS) and the peripheral nervous system (PNS) [10]. They are subdivided into two main classes: muscarinic and nicotinic receptors [11]. Muscarinic AChRs (mAChRs) are G protein-coupled receptors found in cardiac and smooth muscle, secretory glands, and neural tissue [12,13,14]. Nicotinic AChRs (nAChRs), by contrast, are ligand-gated ion channels responsive to both ACh and nicotine. They modulate excitability in skeletal muscle (N1 subtype) and neurons (N2 subtype), with neuronal nAChRs located on postganglionic neuron cell bodies and muscular nAChRs situated at the NMJ on muscle cell surfaces, where they are essential for voluntary motor control [11,15]. Carbachol, a structural analog of ACh, functions as an agonist for both receptor types [16,17,18].

These cholinergic mechanisms are conserved in invertebrates. In the nematode *Caenorhabditis elegans* (*C. elegans*), NMJs are also cholinergic, and many of the proteins involved, including AChRs, are highly conserved across species [19,20,21]. In *C. elegans*, nAChRs at the NMJ are pharmacologically divided into levamisole-sensitive and nicotine-sensitive subtypes [22,23,24], both of which regulate locomotor behavior [22]. One crucial subunit of the nicotine-sensitive class is ACR-16, which closely resembles the vertebrate α7 subunit group [25,26,27]. Mutants lacking *acr-16* have been employed in studies examining nicotine dependence [28].

The *C. elegans* nervous system is composed of only 302 neurons with a completely mapped connectome [29]. Its simplicity, along with low maintenance costs, short generation times, and ethical advantages, makes *C. elegans* an appealing model for high-throughput behavioral studies [30,31]. Locomotor behavior in *C. elegans* is straightforward to quantify and serves as a rapid readout of nervous system function [32,33,34]. Nicotine is known to affect several behaviors in *C. elegans*, such as head thrashing, pharyngeal pumping, egg-laying, and locomotion [22,31,35,36,37,38].

We have previously demonstrated that rESW exposure leads to reversible behavioral changes in *C. elegans*, including reduced locomotion speed and increased paralysis in a dose-dependent manner [39]. These findings support the use of *C. elegans* as a powerful model organism for studying how rESWs interact with AChRs at the NMJ. Moreover, it offers an ethical and economical alternative to vertebrate models, which are limited by higher costs, reduced fecundity, longer generation times, and stricter ethical regulations.

In the present study, we hypothesize that (i) exposure of *C. elegans* to rESWs reduces the effects of nicotine and carbachol on locomotor behavior; and (ii) these effects are mediated via nAChRs at the NMJ in *C. elegans*.

In line with our previous work investigating the behavioral effects of rESWs in *C. elegans* [39,40], worms in this study were maintained in liquid culture.

By elucidating how rESWs modulate neuromuscular transmission via nAChRs in *C. elegans*, this study aims to contribute mechanistic insights relevant to the therapeutic use of rESWT for spasticity in individuals with CP.

## 2. Materials and Methods

### 2.1. Nematodes

N2 wild-type and RB918 *acr-16(ok789)* mutant *C. elegans*, as well as OP50 *Escherichia coli* (*E. coli*), were obtained from the Caenorhabditis Genetics Center (Minneapolis, MN, USA). Note: the RB918 strain was provided by the *C. elegans* Gene Knockout Project at the Oklahoma Medical Research Foundation (Oklahoma City, OK, USA), which was part of the International *C. elegans* Gene Knockout Consortium [41].

### 2.2. Liquid Cultures

Stock *C. elegans* liquid cultures were maintained in 250 mL S Medium supplemented with 0.1% Tween 20 in 500 mL baffled flasks at 20 °C in a shaking incubator (250 rpm; New Brunswick Innova 40, Eppendorf, Hamburg, Germany) [42]. Worms were fed with pelleted *E. coli* OP50.

To prepare synchronized, young adult *C. elegans* for behavioral assays, cultures containing a high proportion of gravid hermaphrodites were selected and transferred into a 500 mL separatory funnel (Thermo Fisher Scientific, Waltham, MA, USA). After approximately 30 min of settling, the first 15 mL were collected into a 15 mL tube and centrifuged at 300× *g* at room temperature for 1 min. Worms were resuspended in 1.5 mL S Medium and subsequently treated with hypochlorite solution for 7–8 min to isolate eggs. After washing and pelleting, the remaining eggs were resuspended in 10 mL of S Medium containing 0.1% Tween 20 and incubated overnight at 20 °C on a rocker platform (30 rpm; PMR-30 Grant-Bio, Grant Instruments, Royston, UK) to reach the L1 larval stage.

On the following day, L1-stage larvae were transferred into 500 mL baffled flasks and maintained in 250 mL S Medium with 0.1% Tween 20 at 24 °C in a shaking incubator (250 rpm). Upon feeding with a pellet of *E. coli* OP50, larvae were allowed to grow for an additional 46 h to reach the young adult stage.

Synchronized young adult worms (>650 µm in length) were harvested by centrifugation (3000× *g*, 4 °C for 5 min). Worms were resuspended in cold NaCl solution and purified using the sucrose floatation method [43]. They were subsequently transferred into S Medium at a concentration of approximately 2 worms/µL. A volume of 10 µL of the worm suspension was dispensed into a single well of a U-bottom 96-well plate (VWR, Radnor, PA, USA), and the well was filled to 300 µL total volume with S Medium for subsequent assaying.

### 2.3. Assays

For each assay, each condition was tested with three technical replicates per experiment, and the entire set of experiments was independently repeated at least three times.

We applied rESW numbers and recovery periods as established in our previous study [39]. Nicotine and carbachol concentrations were selected within previously reported ranges [18,38], and dose–response experiments were conducted to substantiate the choice. Therefore, N2 wild-type worms were placed into a well containing S Medium with or without nicotine (0, 0.5, 2, 10, 20 mM) or carbachol (0, 1, 5, 10 mM). After one hour, the samples were rapidly transferred to food-free nematode growth medium (NGM) plates for video analysis. The results are provided in the Supplementary Materials (Figures S1 and S2).

Based on these conditions, the following assays were performed (Table 1):

Assays A1-2, B1-2, C1,4,7,10,13,16, D1-6 and E1-6: *C. elegans* N2 wild-type or *acr-16* mutant worms were placed into a well containing S Medium with or without nicotine or carbachol, and were immediately exposed to a defined number of rESWs (0, 100 or 500 rESWs, respectively). One hour after the beginning of rESW exposure, samples were rapidly transferred to food-free NGM plates for video analysis.

Assays C2, 3, 5, 6, 8, 9, 11, 12, 14, 15, 17, 18: N2 wild-type worms were exposed to a defined number of rESWs (0, 100 or 500 rESWs, respectively). Following rESW exposure, worms were allowed to recover in the well for a designated time (30 or 180 min, respectively). Subsequently, nicotine was added for one hour, after which worms were rapidly transferred to food-free NGM plates for video analysis.

### 2.4. Application of rESWs

For the application of rESWs, the handpiece of a rESWT device (Swiss DolorClast; Electro Medical Systems, Nyon, Switzerland), equipped with a 6 mm applicator and inner 6 × 2 O-rings (Vi 975, G/FKM 75, C. Otto Gehrckens, Pinneberg, Germany), was mounted on a vertical drill stand (Wolfcraft, Kempenich, Germany). A 5.5 × 2 O-ring (Vi 670/FKM 80, C. Otto Gehrckens) was positioned around the applicator tip, and the handpiece was lowered into a single well of a 96-well plate until the well plate was held firmly in place [39]. A specified number of rESWs was delivered at 5 Hz and 2 Bar, corresponding to an energy flux density of approximately 0.016 mJ/mm^2^ at a 5 mm distance from the applicator.

### 2.5. Transfer and Video Capture

Following the designated waiting period, *C. elegans* liquid samples underwent a rapid transfer procedure for video analysis on NGM plates [39]. To prevent adherence of worms to pipette tips, 30 µL of S Medium containing 1% Tween 20 was added to the well. The well contents were then gently pipetted and released onto a membrane (Polyethersulfone Millipore Express PLUS Membrane, 47 mm diameter, 0.22 µm pore size; Millipore, Billerica, MA, USA) positioned on a vacuum filtration apparatus. The membrane was subsequently inverted onto an NGM plate (without an *E. coli* lawn) and removed just prior to video analysis.

### 2.6. Locomotion Data Collection, Processing and Analysis

For each included worm, the following behavioral parameters were analyzed (Table 2): absolute peristaltic speed, wavelength, number of reversals and number of omega bends. For peristaltic speed and wavelength, average values across all tracked frames were calculated. Reversal and omega bend frequencies were quantified as the percentage of total frames in which these behaviors occurred.

Behavioral video recording of worms on agar plates after rapid transfer was performed using the WormLab Imaging System (MBF Bioscience, Williston, VT, USA). Plates were positioned to ensure that worms were within the objective’s field of view (AF 60 mm 1:2.8D Micro; Nikon, Tokyo, Japan). Video capture was performed using a 20-megapixel monochrome digital camera (acA5472-17um; Basler, Ahrensburg, Germany) at 7.5 frames per second for a duration of 60 s (i.e., 450 frames per video), using WormLab software (Version 2022.1.1, MBF Bioscience). The resolution of each video was 5484 × 3660 pixels. For each sample, tracking was performed across frames 1 to 450.

After tracking, data were exported from WormLab into Microsoft Excel 2010 (Microsoft, Redmond, WA, USA) for further analysis. Only worms with body lengths greater than 650 µm that were continuously tracked for at least 30 s (i.e., ≥225 of 450 frames) were included in the final analysis. Tracks that did not meet these criteria—due to incomplete tracking, segmentation errors or insufficient duration—were excluded. Outliers were identified through visual inspection and removed only when they clearly resulted from tracking artifacts. No data points were excluded solely based on their deviation from the group distribution.

### 2.7. Statistical Analysis

Statistical analysis of all raw data was conducted using GraphPad Prism (version 10.4.1 for Windows; GraphPad Software, San Diego, CA, USA). One-way analysis of variance (ANOVA) was employed to assess statistical differences among groups. For post hoc pairwise comparisons, Bonferroni multiple comparison tests were applied. Details of the statistical analysis for all assays are provided in the Supplementary Materials (Tables S2, S4 and S7). All data were reported as the percentage (%) of the control group corresponding to each respective assay (Table 1). In addition, the Supplementary Materials (Tables S1, S3, S5 and S6) include a summary of absolute mean values, percent of control, corresponding 95% confidence intervals and the total number of worms analyzed per condition. Results are expressed as the arithmetic mean ± standard error of the mean (SEM), based on a minimum of three independent experiments. A *p*-value of <0.05 was considered statistically significant.

## 3. Results

**Figure 1:**
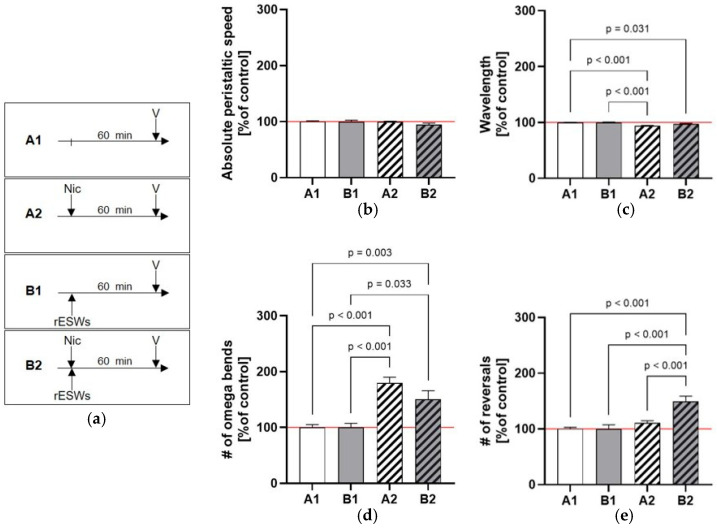
Effects of nicotine and radial extracorporeal shock wave (rESW) exposure on *C. elegans* locomotor behavior. (**a**) Timelines of the different assays as outlined in detail in Table 1 (Nic, nicotine; V, video analysis; rESWs, radial extracorporeal shock waves). (**b**) Mean absolute peristaltic speed was not significantly affected by exposure to 2 mM nicotine alone or in combination with 100 rESWs compared to control worms or those exposed to rESWs alone. (**c**,**d**) Differences in mean wavelength and number of omega bends were observed between nicotine-treated worms and controls, but no significant differences were found between nicotine-treated worms with or without rESW exposure. (**e**) The mean number of reversals remained unchanged following nicotine exposure alone; however, combined treatment with 2 mM nicotine and 100 rESWs significantly increased the number of reversals compared to control worms or those exposed to rESWs alone, suggesting altered locomotor orientation. The data are presented as mean ± SEM; statistical significance was determined as indicated in Section 2. Abbreviations: #, number.

N2 wild-type worms exposed to 2 mM nicotine (Assay A2; hereafter: _A_A2; Figure 1a) did not show significant differences in mean absolute peristaltic speed compared to control worms (_A_A1) (Figure 1b; Tables S1 and S2).

Similarly, the combination of 2 mM nicotine and 100 rESWs (_A_B2) did not significantly affect peristaltic speed relative to control worms (_A_A1) or exposure to 100 rESWs alone (_A_B1) (Figure 1b; Tables S1 and S2). Minor differences in mean wavelength and the number of omega bends were observed between nicotine-treated worms and controls (_A_A2 vs. _A_A1), but not between nicotine-treated worms with or without rESW exposure (_A_B2 vs. _A_A2) (Figure 1c,d; Tables S1 and S2). The mean number of reversals was unaffected by nicotine alone (_A_A2 vs. _A_A1; Figure 1e). However, combined exposure to 2 mM nicotine and 100 rESWs (_A_B2) significantly increased the number of reversals compared to _A_A1, _A_A2, and _A_B1 (Figure 1e), indicating disoriented locomotor behavior. Together, these findings suggest that the combination of 2 mM nicotine and 100 rESWs caused only modest behavioral changes in *C. elegans*.

**Figure 2:**
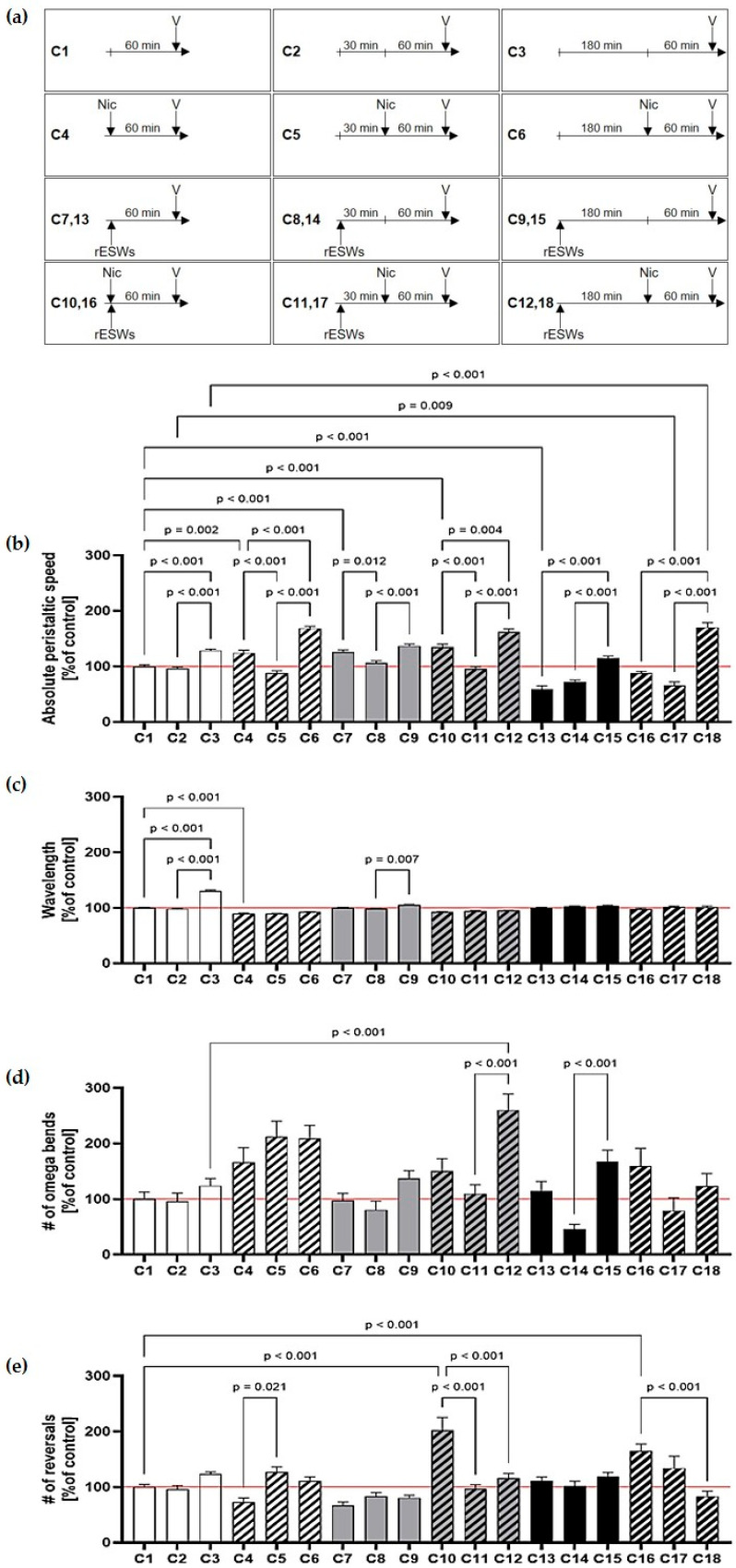
Effects of nicotine, radial extracorporeal shock waves (rESWs), and recovery time on *C. elegans* locomotor behavior. (**a**) Timelines of the different assays as outlined in detail in Table 1 (Nic, nicotine; V, video analysis; rESWs, radial extracorporeal shock waves). (**b**) rESW effects on the mean absolute peristaltic speed were dose- and recovery-dependent: 100 rESWs increased, 500 rESWs decreased speed, and nicotine abolished both effects. After 30 min recovery, speed was reduced, whereas after 180 min recovery, speed increased, particularly with nicotine. (**c**,**d**) Wavelength and omega bends remained largely unchanged. (**e**) Reversals increased without recovery but normalized after 180 min. The data are presented as mean ± SEM; statistical significance was determined as indicated in Section 2. Abbreviations: #, number.

We next examined whether increasing recovery periods after rESW exposure influenced behavioral outcomes in N2 wild-type worms, and whether these effects were modulated by subsequent nicotine treatment (Figure 2a). Without a recovery period, nicotine alone led to a slight increase in mean peristaltic speed, while rESW exposure produced dose-dependent effects (Figure 2b; Tables S3 and S4).

Specifically, 100 rESWs increased peristaltic speed (_A_C7 vs. _A_C1), whereas 500 rESWs decreased it (_A_C13 vs. _A_C1). Notably, the addition of 2 mM nicotine abolished both of these rESW-induced effects (_A_C10 vs. _A_C4; _A_C16 vs. _A_C4; Figure 2b; Tables S3 and S4).

Recovery time following rESW exposure revealed a paradoxical pattern: after 30 min, worms treated with rESWs, with or without nicotine, exhibited significantly reduced peristaltic speeds compared to those without recovery (_A_C5 vs. _A_C4; _A_C8 vs. _A_C7; _A_C11 vs. _A_C10; Figure 2b). In contrast, after 180 min, rESW-treated worms without nicotine showed significantly increased peristaltic speeds compared to those without recovery (_A_C3 vs. _A_C1; _A_C15 vs. _A_C13; Figure 2b). This effect was further enhanced by nicotine (_A_C6 vs. _A_C4; _A_C12 vs. _A_C10; _A_C18 vs. _A_C16; Figure 2b; Tables S3 and S4), suggesting complex time-dependent interactions between nicotine and rESWs.

No systematic differences in wavelength or omega bend frequency were observed across assays, indicating generally coordinated movement (Figure 2c,d; Tables S3 and S4). Consistent with Assays A and B, neither rESWs nor nicotine alone significantly affected the number of reversals when no recovery period was provided (Figure 2e; Tables S3 and S4). However, worms exposed to both rESWs and nicotine without recovery showed significantly more reversals than those with a 180 min recovery (_A_C10 vs. _A_C12; _A_C18 vs. _A_C16; Figure 2e; Tables S3 and S4), further supporting transient disorientation that normalized over time.

**Figure 3:**
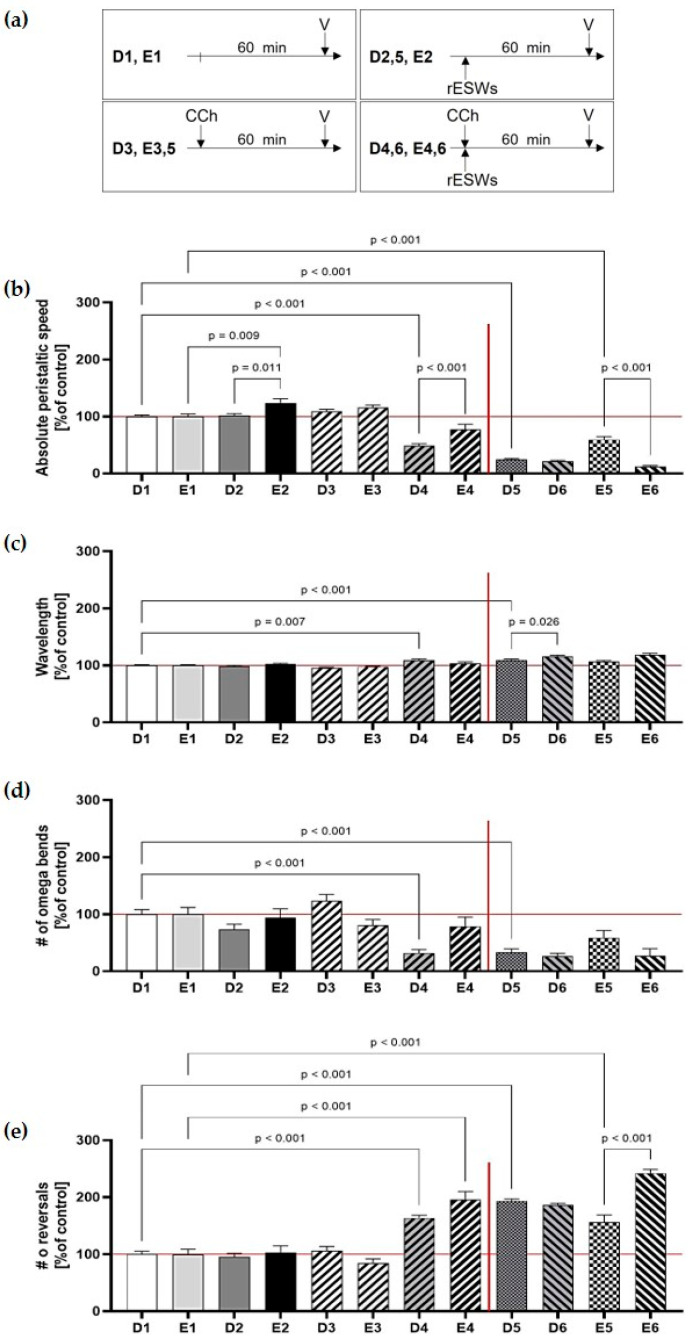
Effects of carbachol and radial extracorporeal shock waves (rESWs) on locomotor behavior of wild-type and *acr-16* mutant *C. elegans*. (**a**) Timelines of the different assays as outlined in detail in Table 1 (CCh, carbachol; V, video analysis; rESWs, radial extracorporeal shock waves). (**b**) 100 rESWs increased speed in *acr-16* mutants, but not in wild-type worms. Carbachol alone had no effect, while its combination with rESWs reduced speed in wild-type worms. (**c**,**d**) No consistent changes were observed in wavelength and omega bends. (**e**) Combined treatment altered reversals. Additional increases were seen with 500 rESWs in wild-type and with 10 mM carbachol in *acr-16* mutants. The data are presented as mean ± SEM; statistical significance was determined as indicated in Section 2. Abbreviations: #, number.

To evaluate the effects of carbachol and rESWs on locomotion, N2 wild-type and *acr-16* mutant worms were exposed to rESWs with or without carbachol (Figure 3a). In *acr-16* mutants, 100 rESWs significantly increased mean peristaltic speed (_A_E2 vs. _A_E1; Figure 3b; Tables S6 and S7), while no such effect was observed in N2 wild-type worms (_A_D2 vs. _A_D1; Figure 3b; Tables S5 and S7).

Carbachol alone (1 mM) had no significant impact on peristaltic speed in either strain (_A_D3 vs. _A_D1; _A_E3 vs. _A_E1; Figure 3b; Table S7). However, the combination of 1 mM carbachol and 100 rESWs significantly reduced peristaltic speed in N2 wild-type worms (_A_D4 vs. _A_D1; Figure 3b; Table S7), but not in *acr-16* mutants (_A_E4 vs. _A_E1; Figure 3b; Table S7), indicating that the observed effects were not solely mediated via NMJs.

Neither strain showed notable changes in wavelength or omega bend frequency after treatment with carbachol, rESWs, or their combination (Figure 3c,d; Table S7). As for reversals, both wild-type and *acr-16* worms displayed a significant increase following combined exposure to 1 mM carbachol and 100 rESWs (_A_D4 vs. _A_D1; _A_E4 vs. _A_E1; Figure 3e; Table S7). Wild-type worms also showed increased reversal frequency after 500 rESWs (_A_D5 vs. _A_D1; Figure 3e; Table S7; not tested in *acr-16* mutants), while *acr-16* mutants exhibited a similar increase after exposure to 10 mM carbachol (_A_E5 vs. _A_E1; Figure 3e; Table S7; not tested in wild-type worms). As in Assays A–C, these results support the conclusion that high rESW intensity or high carbachol concentration induced disoriented locomotor behavior in *C. elegans*.

## 4. Discussion

Radial extracorporeal shock wave therapy (rESWT) is a clinically validated, non-invasive intervention used to manage spasticity in individuals with CP [2,3,4]. Previous work from our group demonstrated (i) the clinical efficacy of rESWT for CP-related spasticity, (ii) rESW-induced reduction and degeneration of AChRs at the NMJ in a rat model [8], and (iii) dose-dependent behavioral effects of rESWs in *C. elegans* [39].

In the present study, we investigated whether rESWs influence the effects of nAChR agonists nicotine and carbachol on *C. elegans* locomotion, using the nematode as a model system. Contrary to our hypothesis, rESWs did not produce significant modulatory effects on nicotine- or carbachol-induced changes in locomotion, nor did they show a specific dependence on nAChRs at the NMJ. Nicotine and carbachol are known to affect worm behavior in a concentration- and time-dependent manner [44]. Nicotine, for example, exerts biphasic effects, enhancing movement at low concentrations and inducing paralysis at high concentrations (>10 mM) [45]. In our study, 2 mM nicotine did not reduce peristaltic speed, suggesting this concentration may have been sub-threshold under the experimental conditions employed—specifically, liquid culture and the method of compound administration. Nevertheless, nicotine altered movement parameters such as wavelength and omega bends, reflecting orientation and foraging behaviors [46,47] and highlighting the influence of environmental context on locomotor assays [48].

Combined exposure to nicotine and rESWs produced comparable locomotor alterations. Our previous work demonstrated that rESWs can induce transient conduction deficits in motor nerves, reduce NMJ AChR density and decrease compound muscle action potentials in rats without causing overt paralysis [8]. In *C. elegans*, although NMJ morphology was not directly assessed, moderate rESW exposure did not disrupt nAChR function sufficiently to significantly alter nicotine-induced locomotion. High numbers of rESWs, however, reduced worm speed in wild-type N2 worms, an effect that attenuated after recovery and further diminished with one hour of nicotine exposure. These observations align with previously reported transient, biphasic and dose-dependent effects of rESWs [9,39,45,49].

Exposure to 1 mM carbachol with rESWs modified locomotion, although carbachol alone had no significant effect, suggesting that rESWs may influence cholinergic signaling beyond the NMJ, possibly influencing additional regions of the nervous system. Importantly, in our model, rESWs were applied in a liquid-based setup that exposes the entire organism uniformly [39]. Given that *C. elegans* is only ~1 mm in length, no shock wave applicators can selectively target peripheral structures, making it very likely that other tissues, including sensory head neurons, are affected. In contrast, clinical and rodent protocols use gel coupling to deliver rESWs locally to specific muscles, tendons or peripheral nerves [4,8,9]. These differences in application likely contribute to divergent mechanistic responses, with whole-body exposure in *C. elegans* reflecting systemic effects rather than isolated PNS modulation.

These results highlight broader implications of rESW action beyond peripheral tissues. While rESWs have not yet been applied to the CNS of vertebrates, our findings in *C. elegans* suggest they may influence CNS-related structures, potentially modulating neural circuits, plasticity or repair processes. Investigating these effects in vertebrate models may clarify underlying mechanisms—such as activity-dependent synaptic modulation, neurogenesis or glial responses—and inform potential therapeutic strategies for neurological disorders.

High rESW doses abolished carbachol-induced behavioral effects in N2 worms, consistent with substantial loss of nAChRs and impaired neurotransmission [8]. Of note, *acr-16* mutants, which lack nicotine-sensitive nAChRs, exhibited locomotion similar to wild-type worms, showing no major baseline deficits while retaining substantial cholinergic postsynaptic currents [27]. In these mutants, 1 mM carbachol alone or combined with rESWs did not alter peristaltic speed, whereas 10 mM carbachol induced locomotor changes amplified by rESWs. These findings suggest potential inhibitory effects of rESWs on nicotine-independent nAChRs and/or mAChRs, with differing effects on wavelength and omega bend frequency further pointing to subtype-specific mechanisms.

Additionally, several off-target pathways may contribute to transient behavioral responses following rESW exposure. Interneurons in the ventral nerve cord and head ganglia, which modulate locomotion and posture without direct synaptic output to muscles, could be affected. Similarly, non-cholinergic systems such as GABAergic motor neurons and glutamatergic circuits may respond to mechanical stress or downstream signaling, altering coordination and linking sensory input to locomotor behavior [50,51]. This underlines the complexity of rESW effects, involving multiple neural subsystems, receptor-specific interactions and both targeted and off-target mechanisms.

In conclusion, consistent with our previous findings [39], rESWs induce transient behavioral changes in *C. elegans*, with responses modulated in complex ways by nAChR agonists. These effects seem to involve neural components analogous to those present in both the CNS and PNS of vertebrates. Since rESWs in *C. elegans* act on the whole organism rather than discrete neuromuscular targets as in vertebrates, the underlying mechanisms may differ from those mediating the therapeutic effects of rESWT in muscle spasticity associated with CP.

While this prevents using *C. elegans* as a model for isolated PNS effects, it also highlights a novel action of rESWs. Importantly, these findings suggest that rESW effects on CNS-related structures in vertebrates might offer new opportunities for developing therapeutic strategies for neurological diseases, highlighting a broader potential that extends beyond their established peripheral targets.

## Figures and Tables

**Table 1:** Assay overview.

Assay	Strain	Chemical	Number of Worms	Concentration [mM]	Number of rESWs	Recovery Period [min]
A1 (control)	N2	nicotine	1309–1311	0	0	-
A2	N2	nicotine	658	2	0	-
B1 (control)	N2	nicotine	265	0	100	-
B2	N2	nicotine	245	2	100	-
C1 (control)	N2	nicotine	343–351	0	0	0
C2	N2	nicotine	174–206	0	0	30
C3	N2	nicotine	307–348	0	0	180
C4	N2	nicotine	97	2	0	0
C5	N2	nicotine	108	2	0	30
C6	N2	nicotine	44	2	0	180
C7	N2	nicotine	175–182	0	100	0
C8	N2	nicotine	216–219	0	100	30
C9	N2	nicotine	250–253	0	100	180
C10	N2	nicotine	107	2	100	0
C11	N2	nicotine	125	2	100	30
C12	N2	nicotine	108	2	100	180
C13	N2	nicotine	273–275	0	500	0
C14	N2	nicotine	174–178	0	500	30
C15	N2	nicotine	208–215	0	500	180
C16	N2	nicotine	90	2	500	0
C17	N2	nicotine	52	2	500	30
C18	N2	nicotine	77	2	500	180
D1 (control)	N2	carbachol	245–257	0	0	-
D2	N2	carbachol	168	0	100	-
D3	N2	carbachol	139–145	1	0	-
D4	N2	carbachol	249	1	100	-
D5	N2	carbachol	406–413	0	500	-
D6	N2	carbachol	398–402	1	500	-
E1 (control)	RB918	carbachol	131	0	0	-
E2	RB918	carbachol	90	0	100	-
E3	RB918	carbachol	179	1	0	-
E4	RB918	carbachol	65	1	100	-
E5	RB918	carbachol	99	10	0	-
E6	RB918	carbachol	136	10	100	-

Abbreviations: rESWs, radial extracorporeal shock waves.

**Table 2:** Summary of endpoint definitions as analyzed by the Wormlab software (MBF Bioscience).

Behavioral Parameter	Definition
Absolute peristaltic speed	Total peristaltic track length (i.e., the sum of forward and reverse movement distances) divided by time, expressed in micrometers per second (µm/s).
Wavelength	Twice the distance between the negative and positive inflection points of the body waveform, measured in micrometers (µm).
Number of omega bends	An omega bend is recorded when the bending angle drops below 90° and continues until the angle exceeds 90° again.
Number of reversals	A reversal is recorded when the worm transitions into backward movement.

## Data Availability

The data that support the findings of this study are available on request from the corresponding author.

## Supplementary Materials

The following supporting information can be downloaded at https://www.mdpi.com/article/10.3390/jcm14207206/s1: Figure S1: Dose–response analysis of nicotine on C. elegans locomotor behavior; Figure S2: Dose–response analysis of carbachol on C. elegans locomotor behavior; Table S1: Summary of absolute mean values of Assays A and B; Table S2: Details of the statistical analysis for Assays A and B; Table S3: Summary of absolute mean values of Assay C; Table S4: Details of the statistical analysis for Assay C; Table S5: Summary of absolute mean values of Assay D; Table S6: Summary of absolute mean values of Assay E; Table S7: Details of the statistical analysis for Assays D and E.

## Abbreviations

The following abbreviations are used in this manuscript:

ACh	acetylcholine
AChR	acetylcholine receptor
CCh	carbachol
*C. elegans*	*Caenorhabditis elegans*
CNS	central nervous system
CP	cerebral palsy
*E. coli*	*Escherichia coli*
mAChR	muscarinic acetylcholine receptor
nAChR	nicotinic acetylcholine receptor
Nic	nicotine
NGM	nematode growth medium
NMJ	neuromuscular junction
PNS	peripheral nervous system
rESW	radial extracorporeal shock wave
rESWT	radial extracorporeal shock wave therapy
SEM	standard error of the mean
