# Supplementary material for: Behavioral Changes in Caenorhabditis elegans After Exposure to Radial Extracorporeal Shock Waves
Source: J Clin Med. 2025 Oct 13;14(20):7206. doi: 10.3390/jcm14207206 (PMC12565115; doi:10.3390/jcm14207206)
Supplement: Supplementary file 1 [file jcm-14-07206-s001.zip › jcm-3682392-supplementary.pdf]

Behavioral Changes in *Caenorhabditis elegans* After Exposure to Radial Extracorporeal Shock Waves

Tanja Hochstrasser [1,*], Leon Kaub [1], Leonard Maier [1], Nicholas B. Angstman [1], Tomonori Kenmoku [2], Carmen Nussbaum-Krammer [1] and Christoph Schmitz [1]

[1] Department of Anatomy II, Ludwig-Maximilians-University of Munich, 81366 Munich, Germany; leon.kaub@med.uni-muenchen.de (L.K.); lpn.maier@gmail.com (L.M.); carmen.nussbaum@med.uni-muenchen.de (C.N.-K.); christoph_schmitz@med.uni-muenchen.de (C.S.)

[2] Department of Orthopedic Surgery, Kitasato University School of Medicine, Sagamihara 252-0329, Japan; kenmoku@med.kitasato-u.ac.jp

* Correspondence: tanja.hochstrasser@med.uni-muenchen.de

Supplementary Materials

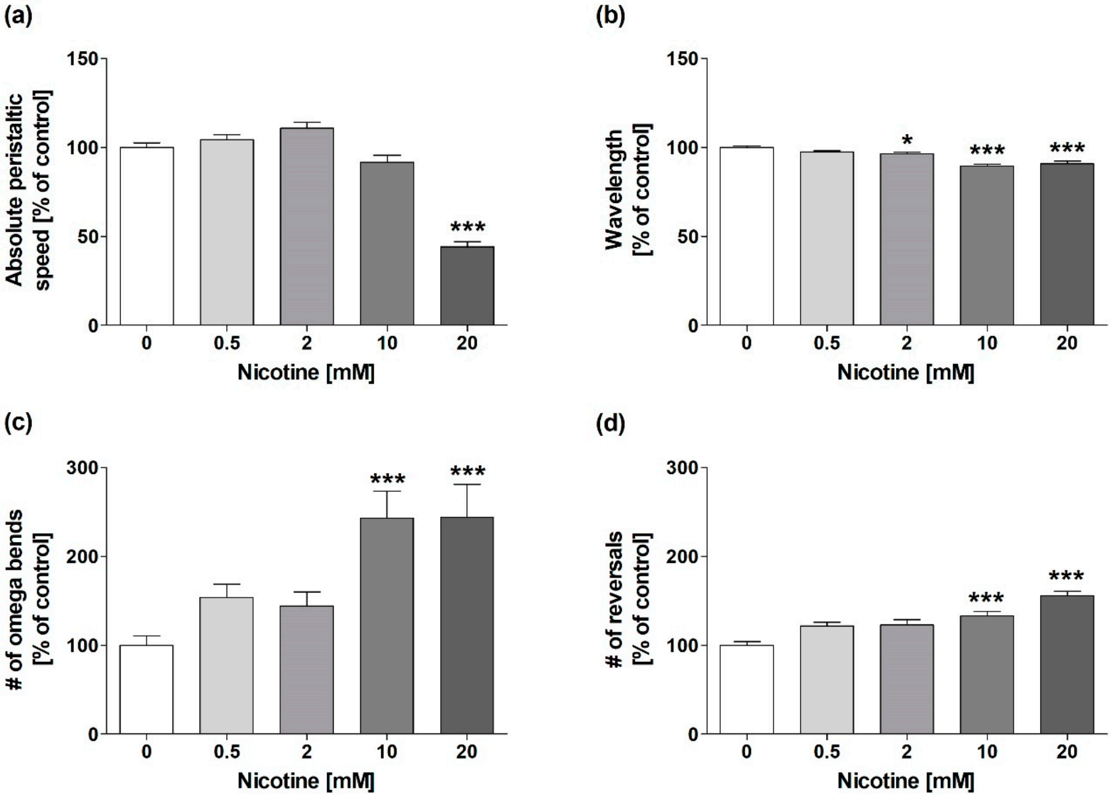

Figure S1. Dose–response analysis of nicotine on *C. elegans* locomotor behavior. Worms were exposed to increasing concentrations of nicotine (0 mM (n=309), 0.5 mM (n=296), 2 mM (n=207), 10 mM (n=190), 20 mM (n=155)). (a) Mean ± SEM of absolute peristaltic speed, (b) wavelength, (e) number of omega bends and (e) number of reversals. Data were analyzed using one-way ANOVA followed by Bonferroni's multiple comparison test (*p < 0.05; **p < 0.01; ***p < 0.001). Statistical significance (indicated by *) is shown relative to controls.

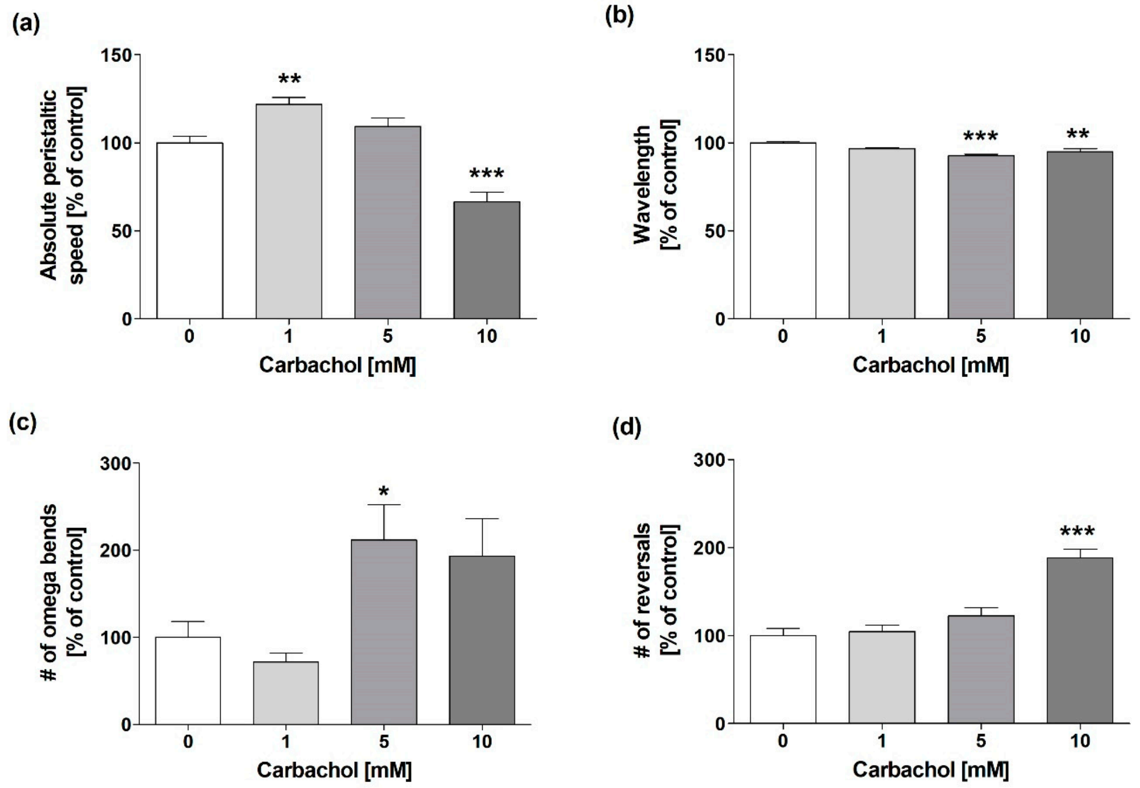

Figure S2. Dose–response analysis of carbachol on *C. elegans* locomotor behavior. Worms were exposed to increasing concentrations of carbachol (0 mM (n=110), 1 mM (n=172), 5 mM (n=111), 10 mM (n=116)). (a) Mean ± SEM of absolute peristaltic speed, (b) wavelength, (e) number of omega bends, and (e) number of reversals. Data were analyzed using one-way ANOVA followed by Bonferroni's multiple comparison test (*p < 0.05; **p < 0.01; ***p < 0.001). Statistical significance (indicated by *) is shown relative to controls.

Table S1. Summary of absolute mean values, % of control, and corresponding 95% confidence intervals (CIs) for absolute peristaltic speed, wavelength, omega bends, and reversal frequency in Assays A and B.

		A1	A2	B1	B2
Absolute peristaltic speed	µm/s	112.00 ± 1.47 (1311)	116.40 ± 1.99 (658)	132.00 ± 3.31 (265)	124.70 ± 3.71(245)
	% of control	100.00 ± 1.34 (1311)	103.90 ± 1.78 (658)	100.00 ± 2.51 (265)	94.45 ± 2.81(245)
	95 % CI (% of control)	97.4 -102.6	100.4 - 107.4	95.1 - 104.9	88.9 - 100.0
Wavelength	µm	352.30 ± 1.35 (1311)	335.70 ± 1.78 (658)	335.50 ± 3.09 (265)	326.50 ± 3.24(245)
	% of control	100.00 ± 0.38 (1311)	94.03 ± 0.50 (658)	100.00 ± 0.92 (265)	97.32 ± 0.97(245)
	95 % CI (% of control)	99.3 - 100.7	93.1 - 95.0	98.2 - 101.8	95.4 - 99.2
Omega bends	average/frame	0.0579 ± 0.0028 (1309)	0.0957 ± 0.0055 (658)	0.0493 ± 0.0061 (265)	0.0742 ± 0.075 (245)
	% of control	100.00 ± 4.87 (1309)	179.90 ± 10.30 (658)	100.00 ± 7.40 (265)	150.60 ± 15.29(245)
	95 % CI (% of control)	90.7 - 109.8	159.7 - 200.1	85.5 - 114.6	120.5 - 180.7
Reversals	average/frame	0.0077 ± 0.0003 (1311)	0.0085 ± 0.0003 (658)	0.0042 ± 0.0003 (265)	0.0064 ± 0.0004 (245)
	% of control	100.00 ± 3.35 (1311)	111.01 ± 3.85 (658)	100.00 ± 7.40 (265)	149.10 ± 9.78 (245)
	95 % CI (% of control)	93.4 - 106.6	103.2 -118.3	85.5 - 114.6	129.9 - 168.4

Data are presented as mean ± SEM (n) for absolute values and % of control.

Table S2. Details of the statistical analysis for Assays A and B, including p values and 95% confidence intervals of differences (CIs of diff) for peristaltic speed, wavelength, omega bends, and reversal frequency.

	Absolut peristaltic speed		Wavelength		Omega bends		Reversals	
P value (ANOVA):	0.3798		< 0.0001		< 0.0001		< 0.0001	
	Bonferroni's Multiple Comparison Test							
	P value	95% CI of diff	P value	95% CI of diff	P value	95% CI of diff	P value	95% CI of diff
**A1 vs A2**	ns	-5,095 to 6,526	***	4,228 to 7,706	***	-105,6 to -53,76	ns	-23,34 to 2,699
**A1 vs B1**	ns	-8,188 to 8,195	ns	-2,452 to 2,451	ns	-36,34 to 36,72	ns	-18,00 to 18,72
**A1 vs B2**	ns	-2,914 to 14,02	*	0,1503 to 5,218	**	-88,10 to -12,61	***	-67,68 to -29,74
**A2 vs B1**	ns	-9,561 to 8,137	***	-8,616 to -3,319	***	40,40 to 119,3	ns	-9,146 to 30,51
**A2 vs B2**	ns	-4,267 to 13,94	**	-6,008 to -0,5587	ns	-11,27 to 69,90	***	-58,78 to -17,99
**B1 vs B2**	ns	-5,232 to 16,33	ns	-0,5418 to 5,911	*	-98,60 to -2,478	***	-73,22 to -24,91

Effects of nicotine or radial extracorporeal shock waves were analyzed using one-way ANOVA followed by Bonferroni's multiple comparison test. *$p < 0.05$; **$p < 0.01$; ***$p < 0.001$; ns, not significant.

Table S3. Summary of absolute mean values, % of control, and corresponding 95% confidence intervals (CIs) for absolute peristaltic speed, wavelength, omega bends, and reversal frequency in Assay C.

		C1	C2	C3	C4	C5	C6
Absolute peristaltic speed	µm/s	106.54 ± 2.94 (351)	102.46 ± 2.89 (206)	137.09 ± 2.49 (348)	132.65 ± 5.37 (97)	93.78 ± 4.43 (108)	179.78 ± 5.29 (44)
	% of control	100.00 ± 2.76 (351)	96.29 ± 2.71 (206)	128.70 ± 2.34 (348)	124.40 ± 5.04 (97)	87.96 ± 4.16 (108)	168.70 ± 4.96 (44)
	95 % CI (% of control)	94.6 – 105.4	91.0 – 101.6	124.1 – 133.3	114.4 – 134.4	79.7 – 96.2	158.7 – 178.7
Wavelength	µm	345.75 ± 2.66 (351)	339.63 ± 3.42 (206)	350.16 ± 3.42 (348)	310.17 ± 3.22 (97)	307.96 ± 3.60 (108)	319.02 ± 3.80 (44)
	% of control	100.00 ± 0.77 (351)	98.23 ± 0.99 (206)	130.20 ± 2.12 (348)	89.71 ± 0.93 (97)	89.07 ± 1.04 (108)	92.27 ± 1.10 (44)
	95 % CI (% of control)	98.6 – 101.6	96.9 – 100.2	126.0 – 134.4	87.9 – 91.55	87.00 – 91.1	90.5 – 94.5
Omega bends	average/frame	0.0468 ± 0.0058 (343)	0.0449 ± 0.0069 (174)	0.0581 ± 0.0060 (307)	0.0780 ± 0.0121 (97)	0.0995 ± 0.0128 (108)	0.0978 ± 0.0155 (44)
	% of control	100.01 ± 12.40 (343)	96.09 ± 14.77 (174)	124.20 ± 12.95 (307)	166.57 ± 25.81 (97)	212.58 ± 27.44 (108)	209.10 ± 33.15 (44)
	95 % CI (% of control)	75.6 - 124.4	66.9 - 125.2	98.9 - 149.7	115.3 - 217.8	158.2 - 267.0	142.2 - 275.9
Reversals	average/frame	0.0079 ± 0.0004 (351)	0.0076 ± 0.0005 (206)	0.0098 ± 0.0003 (348)	0.0058 ± 0.0006 (97)	0.0100 ± 0.0007 (108)	0.0088 ± 0.0008 (44)
	% of control	100.00 ± 4.77(351)	96.15 ± 6.55 (206)	123.80 ± 3.45 (348)	72.89 ± 7.81 (97)	127.06 ± 9.11 (108)	111.50 ± 9.53 (44)
	95 % CI (% of control)	90.6 - 109.4	83.2 - 109.1	117.0 - 130.6	47.4 - 88.4	109.0 - 145.1	92.2 - 130.7

		C7	C8	C9	C10	C11	C12
Absolute peristaltic speed	µm/s	134.37 ± 4.09 (182)	113.44 ± 3.77 (219)	145.69 ± 3.34 (253)	143.40 ± 5.91 (107)	101.71 ± 4.45 (125)	173.45 ± 5.31 (108)
	% of control	125.90 ± 3.84 (182)	106.50 ± 3.54 (219)	136.90 ± 3.13 (253)	134.70 ± 5.55 (107)	95.45 ± 4.18 (125)	162.70 ± 4.98 (108)
	95 % CI (% of control)	118.3 – 133.5	99.5 – 113.4	130.7 – 143.1	123.7 – 145.7	87.12 – 103.7	152.8 – 172.6
Wavelength	µm	346.58 ± 3.32 (182)	339.73 ± 3.25 (219)	364.18 ± 2.90 (253)	320.27 ± 3.87 (107)	327.01 ± 3.18 (125)	328.32 ± 3.11 (108)
	% of control	100.24 ± 0.96 (182)	98.26 ± 0.94 (219)	105.33 ± 0.84 (253)	92.63 ± 1.12 (107)	94.58 ± 0.92 (125)	94.96 ± 0.90 (108)
	95 % CI (% of control)	98.3 – 102.1	96.4 – 100.1	103.7 – 107.0	90.4 – 94.8	92.8 – 96.4	93.2 – 96.8
Omega bends	average/frame	0.0457 ± 0.0059 (175)	0.0377 ± 0.0073 (216)	0.0641 ± 0.0067 (250)	0.0703 ± 0.0110 (107)	0.0515 ± 0.0075 (125)	0.1219 ± 0.0135 (108)
	% of control	97.58 ± 12.54 (175)	80.64 ± 15.62 (216)	136.91 ± 14.28 (250)	150.20 ± 23.60 (107)	109.99 ± 16.01 (125)	260.41 ± 28.87 (108)
	95 % CI (% of control)	72.8 - 122.3	49.8 - 111.4	108.8 - 165.0	105.4 - 194.9	78.3 - 141.7	203.2 - 317.6
Reversals	average/frame	0.0052 ± 0.0005 (182)	0.0066 ± 0.0005 (219)	0.0064 ± 0.0004 (253)	0.0160 ± 0.0018 (107)	0.0077 ± 0.0006 (125)	0.0092 ± 0.0007 (108)
	% of control	66.85 ± 6.34 (182)	83.82 ± 5.99 (219)	80.58 ± 4.71 (253)	202.30 ± 23.03 (107)	96.80 ± 7.41 (125)	115.83 ± 9.15 (108)
	95 % CI (% of control)	54.3 - 79.4	72.0 - 95.6	71.3 - 89.9	156.6 - 248.0	82.1 - 111.5	97.7 - 134.0

		C13	C14	C15	C16	C17	C18
Absolute peristaltic speed	µm/s	62.47 ± 6.55 (275)	77.29 ± 3.36 (178)	122.56 ± 3.48 (215)	94.06 ± 3.18 (90)	70.01 ± 6.78 (52)	181.50 ± 8.76 (77)
	% of control	58.67 ± 6.14 (275)	72.57 ± 3.15 (178)	115.20 ± 3.26 (215)	88.21 ± 2.98 (90)	65.66 ± 6.36 (52)	170.40 ± 8.22 (77)
	95 % CI (% of control)	46.5 – 70.9	66.4 – 78.8	108.8 – 121.6	82.3 – 94.08	52.9 – 78.4	154.0 – 186.8
Wavelength	µm	346.78 ± 3.32 (275)	354.94 ± 4.29 (178)	359.09 ± 4.01 (215)	338.18 ± 6.05 (90)	351.04 ± 6.71 (52)	350.90 ± 9.92 (77)
	% of control	100.30 ± 0.96 (275)	102.66 ± 1.24 (178)	103.86 ± 1.16 (215)	97.81 ± 1.75 (90)	101.53 ± 1.94 (52)	101.49 ± 2.00 (77)
	95 % CI (% of control)	98.4 – 102.2	100.2 – 105.1	101.5 – 106.0	94.3 – 101.3	97.6 – 105.4	97.5 – 105.5
Omega bends	average/frame	0.0537 ± 0.0079 (273)	0.0214 ± 0.0042 (174)	0.0782 ± 0.0097 (208)	0.0746 ± 0.0150 (90)	0.0369 ± 0.0109 (52)	0.0578 ± 0.0105 (70)
	% of control	114.81 ± 16.93 (273)	45.75 ± 8.91 (174)	167.10 ± 20.75 (208)	159.33 ± 32.08 (90)	78.91 ± 23.29 (52)	123.52 ± 22.41 (70)
	95 % CI (% of control)	81.5 - 148.1	28.2 - 63.3	126.2 - 208.0	95.6 - 223.1	32.2 - 125.7	78.9 - 168.1
Reversals	average/frame	0.0088 ± 0.0005 (275)	0.0081 ± 0.0007 (178)	0.0094 ± 0.0006 (215)	0.0130 ± 0.0010 (90)	0.0106 ± 0.0017 (52)	0.0066 ± 0.0008 (77)
	% of control	111.36 ± 6.50 (275)	101.92 ± 9.08 (178)	118.90 ± 7.32 (215)	164.90 ± 12.77 (90)	134.17 ± 21.52 (52)	83.18 ± 9.59 (77)
	95 % CI (% of control)	98.6 - 124.2	84.0 - 119.8	104.5 - 133.3	139.5 - 190.3	90.9 -177.4	64.1 - 102.3

Data are presented as mean ± SEM (n) for absolute values and % of control.

Table S4. Details of the statistical analysis for Assay C, including p values and 95% confidence intervals of differences (CIs of diff) for peristaltic speed, wavelength, omega bends, and reversal frequency.

	Absolut peristaltic speed		Wavelength		Omega bends		Reversals	
P value (ANOVA):	< 0.0001		< 0.0001		< 0.0001		< 0.0001	
	Bonferroni's Multiple Comparison Test							
	P value	95% CI of diff	P value	95% CI of diff	P value	95% CI of diff	P value	95% CI of diff
**C1 vs C2**	ns	-11.80 to 19.22	ns	-4.110 to 7.793	ns	-73.82 to 81.66	ns	-28.33 to 36.02
**C1 vs C3**	***	-42.09 to -15.36	***	-35.25 to -24.99	ns	-102.2 to 53.87	ns	-51.55 to 3.921
**C1 vs C4**	**	-44.65 to -4.113	***	2.583 to 18.14	ns	-162.6 to 29.49	ns	-14.95 to 69.16
**C1 vs C5**	ns	-7.402 to 31.48	***	3.547 to 18.47	**	-204.7 to -20.41	ns	-67.40 to 13.28
**C1 vs C6**	***	-96.99 to -40.48	ns	-3.038 to 18.65	ns	-242.8 to 24.71	ns	-70.09 to 47.17
**C1 vs C7**	***	-42.03 to -9.755	ns	-6.361 to 6.027	ns	-75.16 to 80.03	ns	-0.3397 to 66.64
**C1 vs C8**	ns	-21.69 to 8.739	ns	-4.022 to 7.656	ns	-53.18 to 91.92	ns	-15.40 to 47.74
**C1 vs C9**	***	-51.46 to -22.32	ns	-10.84 to 0.3406	ns	-106.4 to 32.56	ns	-10.82 to 49.65
**C1 vs C10**	***	-54.18 to -15.15	ns	-0.04343 to 14.93	ns	-142.6 to 42.35	***	-142.8 to -61.83
**C1 vs C11**	ns	-13.86 to 22.95	ns	-1.571 to 12.55	ns	-97.24 to 77.29	ns	-34.99 to 41.38
**C1 vs C12**	***	-82.13 to -43.24	ns	-2.349 to 12.57	***	-252.6 to -68.24	ns	-56.18 to 24.50
**C1 vs C13**	***	20.46 to 62.21	ns	-5.683 to 5.239	ns	-82.55 to 52.94	ns	-40.89 to 18.16
**C1 vs C14**	***	11.17 to 43.69	ns	-8.827 to 3.652	ns	-23.48 to 132.0	ns	-35.66 to 31.81
**C1 vs C15**	ns	-30.48 to 0.1257	ns	-9.564 to 2.181	ns	-140.5 to 6.291	ns	-50.67 to 12.83
**C1 vs C16**	ns	-2.435 to 26.02	ns	-5.750 to 10.27	ns	-158.2 to 39.61	***	-108.2 to -21.59
**C1 vs C17**	***	8.088 to 60.60	ns	-11.53 to 8.618	ns	-103.2 to 145.4	ns	-88.65 to 20.30
**C1 vs C18**	***	-92.63 to -48.16	ns	-9.951 to 7.115	ns	-128.8 to 81.82	ns	-29.32 to 62.95
**C2 vs C3**	***	-47.96 to -16.90	***	-37.92 to -26.00	ns	-117.9 to 61.71	ns	-59.89 to 4.570
**C2 vs C4**	***	-49.85 to -6.331	*	0.1701 to 16.87	ns	-176.3 to 35.36	ns	-21.89 to 68.40
**C2 vs C5**	ns	-12.66 to 29.32	**	1.111 to 17.22	**	-218.8 to -14.18	ns	-74.46 to 12.64
**C2 vs C6**	***	-101.8 to -43.10	ns	-5.297 to 17.23	ns	-253.9 to 27.98	ns	-76.19 to 45.58
**C2 vs C7**	***	-47.58 to -11.63	ns	-8.907 to 4.889	ns	-90.91 to 87.94	ns	-7.994 to 66.60
**C2 vs C8**	ns	-27.33 to 6.966	ns	-6.606 to 6.557	ns	-69.64 to 100.5	ns	-23.26 to 47.91
**C2 vs C9**	***	-57.18 to -24.02	**	-13.46 to -0.7295	ns	-123.3 to 41.64	ns	-18.84 to 49.97
**C2 vs C10**	***	-59.43 to -17.32	ns	-2.477 to 13.68	ns	-156.7 to 48.55	***	-149.8 to -62.47
**C2 vs C11**	ns	-19.19 to 20.87	ns	-4.038 to 11.34	ns	-111.8 to 84.04	ns	-42.22 to 40.91
**C2 vs C12**	***	-87.38 to -45.40	ns	-4.784 to 11.33	***	-266.6 to -62.00	ns	-63.24 to 23.87
**C2 vs C13**	***	15.30 to 59.95	ns	-8.312 to 4.185	ns	-99.75 to 62.30	ns	-48.99 to 18.57
**C2 vs C14**	***	5.639 to 41.80	ns	-11.37 to 2.510	ns	-39.21 to 139.9	ns	-43.29 to 31.75
**C2 vs C15**	*	-36.11 to -1.657	ns	-12.14 to 1.078	ns	-156.8 to 14.78	ns	-58.51 to 12.98
**C2 vs C16**	ns	-8.194 to 24.37	ns	-8.148 to 8.988	ns	-171.7 to 45.21	***	-115.1 to -22.43
**C2 vs C17**	**	3.214 to 58.06	ns	-13.82 to 7.224	ns	-114.8 to 149.2	ns	-94.92 to 18.87
**C2 vs C18**	***	-97.70 to -50.50	ns	-12.32 to 5.798	ns	-141.8 to 86.90	ns	-36.00 to 61.94
**C3 vs C4**	ns	-15.94 to 24.63	***	32.70 to 48.27	ns	-148.4 to 63.67	**	8.822 to 93.01
**C3 vs C5**	***	21.30 to 60.23	***	33.66 to 48.60	ns	-190.9 to 14.15	ns	-43.63 to 37.13
**C3 vs C6**	***	-68.28 to -11.74	***	27.08 to 48.78	ns	-226.0 to 56.24	ns	-46.30 to 71.01
**C3 vs C7**	ns	-13.33 to 18.99	***	23.75 to 36.16	ns	-63.07 to 116.3	***	23.42 to 90.50
**C3 vs C8**	***	7.009 to 37.49	***	26.09 to 37.79	ns	-41.81 to 128.9	***	8.363 to 71.61
**C3 vs C9**	ns	-22.76 to 6.432	***	19.27 to 30.47	ns	-95.47 to 70.02	***	12.94 to 73.52
**C3 vs C10**	ns	-25.47 to 13.59	***	30.07 to 45.06	ns	-128.8 to 76.87	***	-119.0 to -37.97
**C3 vs C11**	***	14.85 to 51.69	***	28.54 to 42.68	ns	-83.97 to 112.4	ns	-11.22 to 65.24
**C3 vs C12**	***	-53.42 to -14.50	***	27.77 to 42.70	***	-238.8 to -33.68	ns	-32.41 to 48.36
**C3 vs C13**	***	49.16 to 90.95	***	24.43 to 35.37	ns	-71.94 to 90.68	ns	-17.13 to 42.03
**C3 vs C14**	***	39.87 to 72.43	***	21.29 to 33.78	ns	-11.37 to 168.2	ns	-11.90 to 55.67
**C3 vs C15**	ns	-1.778 to 28.88	***	20.55 to 32.31	ns	-129.0 to 43.14	ns	-26.91 to 36.69
**C3 vs C16**	***	26.26 to 54.77	***	24.36 to 40.40	ns	-143.8 to 73.52	ns	-84.44 to 2.264
**C3 vs C17**	***	36.80 to 89.34	***	18.58 to 38.75	ns	-86.91 to 177.5	ns	-64.87 to 44.14
**C3 vs C18**	***	-63.92 to -19.42	***	20.16 to 37.24	ns	-113.9 to 115.2	ns	-5.545 to 86.80
**C4 vs C5**	***	11.70 to 61.14	ns	-8.839 to 10.13	ns	-162.9 to 70.83	*	-105.5 to -2.881
**C4 vs C6**	***	-76.47 to -12.24	ns	-14.88 to 9.770	ns	-194.3 to 109.3	ns	-105.2 to 28.08
**C4 vs C7**	ns	-23.72 to 20.70	**	-19.05 to -2.004	ns	-36.73 to 174.7	ns	-40.04 to 52.13

**C4 vs C8**	ns	-3.645 to 39.46	*	-16.82 to -0.2745	ns	-16.16 to 188.0	ns	-55.64 to 33.78
**C4 vs C9**	ns	-33.61 to 8.591	***	-23.71 to -7.515	ns	-70.26 to 129.6	ns	-51.47 to 36.09
**C4 vs C10**	ns	-35.06 to 14.49	ns	-12.42 to 6.590	ns	-100.7 to 133.5	***	-180.8 to -78.02
**C4 vs C11**	**	5.019 to 52.83	ns	-14.05 to 4.305	ns	-56.44 to 169.6	ns	-73.52 to 25.70
**C4 vs C12**	***	-63.02 to -13.59	ns	-14.73 to 4.237	ns	-210.7 to 23.00	ns	-94.22 to 8.344
**C4 vs C13**	***	39.86 to 91.58	***	-18.59 to -2.576	ns	-46.97 to 150.5	ns	-81.76 to 4.826
**C4 vs C14**	***	29.51 to 74.11	***	-21.51 to -4.392	**	14.98 to 226.7	ns	-75.30 to 17.24
**C4 vs C15**	ns	-12.41 to 30.82	***	-22.35 to -5.759	ns	-103.2 to 102.1	*	-90.86 to -1.181
**C4 vs C16**	***	15.31 to 57.04	ns	-18.02 to 1.825	ns	-115.0 to 129.5	***	-145.7 to -38.35
**C4 vs C17**	***	28.36 to 89.09	*	-23.47 to -0.1650	ns	-55.90 to 231.2	ns	-124.3 to 1.732
**C4 vs C18**	***	-72.98 to -19.04	**	-22.13 to -1.430	ns	-84.43 to 170.5	ns	-66.25 to 45.67
**C5 vs C6**	***	-112.4 to -49.17	ns	-15.33 to 8.925	ns	-145.9 to 152.9	ns	-49.96 to 81.17
**C5 vs C7**	***	-59.40 to -16.47	***	-19.41 to -2.939	**	12.80 to 217.2	***	15.68 to 104.7
**C5 vs C8**	ns	-39.29 to 2.260	**	-17.16 to -1.218	***	33.51 to 230.4	*	0.1283 to 86.34
**C5 vs C9**	***	-69.24 to -28.62	***	-24.05 to -8.466	ns	-20.51 to 171.9	*	4.339 to 88.62
**C5 vs C10**	***	-70.81 to -22.61	ns	-12.81 to 5.686	ns	-51.50 to 176.4	***	-125.3 to -25.24
**C5 vs C11**	ns	-30.71 to 15.72	ns	-14.43 to 3.392	ns	-7.132 to 212.3	ns	-17.91 to 78.42
**C5 vs C12**	***	-98.77 to -50.68	ns	-15.12 to 3.332	ns	-161.5 to 65.84	ns	-38.66 to 61.12
**C5 vs C13**	**	4.077 to 54.51	***	-18.93 to -3.530	*	2.823 to 192.7	ns	-25.93 to 57.33
**C5 vs C14**	ns	-6.164 to 36.94	***	-21.87 to -5.325	***	64.52 to 269.2	ns	-19.58 to 69.85
**C5 vs C15**	***	-48.06 to -6.377	***	-22.70 to -6.702	ns	-53.60 to 144.5	ns	-35.10 to 51.38
**C5 vs C16**	ns	-20.31 to 19.82	ns	-18.42 to 0.9316	ns	-65.95 to 172.5	ns	-90.16 to 14.49
**C5 vs C17**	ns	-7.521 to 52.13	*	-23.91 to -1.021	ns	-7.308 to 274.7	ns	-68.99 to 54.77
**C5 vs C18**	***	-108.8 to -56.08	**	-22.54 to -2.313	ns	-35.51 to 213.6	ns	-10.80 to 98.56
**C6 vs C7**	***	13.16 to 72.52	ns	-19.37 to 3.418	ns	-29.38 to 252.3	ns	-16.98 to 106.2
**C6 vs C8**	***	33.07 to 91.45	ns	-17.19 to 5.213	ns	-9.734 to 266.6	ns	-32.94 to 88.20
**C6 vs C9**	*	2.983 to 60.70	**	-24.13 to -1.982	ns	-64.40 to 208.7	ns	-29.01 to 90.75
**C6 vs C10**	*	2.425 to 65.71	ns	-12.51 to 11.78	ns	-90.68 to 208.5	***	-156.5 to -25.20
**C6 vs C11**	***	42.31 to 104.3	ns	-14.20 to 9.572	ns	-47.34 to 245.5	ns	-49.61 to 78.92
**C6 vs C12**	ns	-25.55 to 37.65	ns	-14.82 to 9.434	ns	-200.7 to 98.03	ns	-69.95 to 61.19
**C6 vs C13**	***	77.57 to 142.6	ns	-19.04 to 2.982	ns	-41.44 to 229.9	ns	-59.44 to 59.62
**C6 vs C14**	***	66.41 to 125.9	ns	-21.81 to 1.023	**	22.37 to 304.3	ns	-52.19 to 71.25
**C6 vs C15**	***	24.32 to 82.79	*	-22.72 to -0.2775	ns	-96.66 to 180.5	ns	-68.12 to 53.20
**C6 vs C16**	***	51.84 to 109.2	ns	-18.02 to 6.929	ns	-103.9 to 203.4	ns	-120.9 to 13.99
**C6 vs C17**	***	66.88 to 139.3	ns	-23.15 to 4.626	ns	-40.94 to 301.2	ns	-97.82 to 52.38
**C6 vs C18**	ns	-35.05 to 31.73	ns	-22.04 to 3.591	ns	-72.31 to 243.4	ns	-41.01 to 97.55
**C7 vs C8**	*	1.696 to 37.14	ns	-4.817 to 8.786	ns	-68.02 to 101.9	ns	-53.75 to 19.80
**C7 vs C9**	ns	-28.17 to 6.176	ns	-11.68 to 1.506	ns	-121.7 to 42.99	ns	-49.37 to 21.90
**C7 vs C10**	ns	-30.30 to 12.75	ns	-0.6485 to 15.87	ns	-155.1 to 49.93	***	-180.1 to -90.80
**C7 vs C11**	***	9.914 to 50.96	ns	-2.218 to 13.54	ns	-110.2 to 85.40	ns	-72.54 to 12.63
**C7 vs C12**	***	-58.25 to -15.33	ns	-2.956 to 13.52	***	-265.0 to -60.63	*	-93.52 to -4.455
**C7 vs C13**	***	44.46 to 90.00	ns	-6.534 to 6.425	ns	-98.12 to 63.65	***	-79.55 to -9.481
**C7 vs C14**	***	34.70 to 71.95	ns	-9.569 to 4.728	ns	-37.59 to 141.2	ns	-73.72 to 3.573
**C7 vs C15**	ns	-7.079 to 28.51	ns	-10.35 to 3.306	ns	-155.2 to 16.13	***	-89.00 to -15.14
**C7 vs C16**	***	20.80 to 54.57	ns	-6.309 to 11.17	ns	-170.1 to 46.59	***	-145.3 to -50.81
**C7 vs C17**	***	32.45 to 88.02	ns	-11.95 to 9.372	ns	-113.3 to 150.6	**	-125.0 to -9.677
**C7 vs C18**	***	-68.52 to -20.48	ns	-10.47 to 7.968	ns	-140.2 to 88.28	ns	-66.17 to 33.51
**C8 vs C9**	***	-46.72 to -14.11	**	-13.33 to -0.8098	ns	-133.9 to 21.32	ns	-30.59 to 37.08
**C8 vs C10**	***	-49.03 to -7.350	ns	-2.370 to 13.63	ns	-168.3 to 29.23	***	-161.7 to -75.24
**C8 vs C11**	ns	-8.785 to 30.83	ns	-3.927 to 11.28	ns	-123.2 to 64.52	ns	-54.08 to 28.12
**C8 vs C12**	***	-76.98 to -35.43	ns	-4.677 to 11.27	***	-278.2 to -81.33	ns	-75.12 to 11.10
**C8 vs C13**	***	25.69 to 69.93	ns	-8.180 to 4.103	ns	-110.2 to 41.89	ns	-60.74 to 5.666
**C8 vs C14**	***	16.07 to 51.73	ns	-11.25 to 2.439	ns	-50.19 to 120.0	ns	-55.10 to 18.90
**C8 vs C15**	ns	-25.66 to 8.263	ns	-12.02 to 1.002	*	-167.6 to -5.340	ns	-70.29 to 0.1053
**C8 vs C16**	**	2.268 to 34.27	ns	-8.045 to 8.935	ns	-183.5 to 26.11	***	-127.0 to -35.17
**C8 vs C17**	***	13.56 to 68.07	ns	-13.74 to 7.186	ns	-127.3 to 130.8	ns	-106.9 to 6.206
**C8 vs C18**	***	-87.33 to -40.51	ns	-12.22 to 5.749	ns	-153.7 to 67.99	ns	-47.93 to 49.21
**C9 vs C10**	ns	-18.15 to 22.60	***	4.877 to 20.52	ns	-109.7 to 83.25	***	-164.0 to -79.45
**C9 vs C11**	***	22.12 to 60.75	***	3.330 to 18.16	ns	-64.57 to 118.4	ns	-56.30 to 23.86
**C9 vs C12**	***	-46.10 to -5.485	***	2.570 to 18.16	***	-219.7 to -27.32	ns	-77.39 to 6.887

**C9 vs C13**	***	56.54 to 99.91	ns	-0.8774 to 10.94	ns	-51.02 to 95.22	ns	-62.72 to 1.156
**C9 vs C14**	***	47.03 to 81.60	ns	-3.970 to 9.298	*	8.701 to 173.6	ns	-57.21 to 14.52
**C9 vs C15**	***	5.326 to 38.10	ns	-4.729 to 7.850	ns	-108.6 to 48.18	**	-72.34 to -4.330
**C9 vs C16**	***	33.29 to 64.08	ns	-0.8091 to 15.84	ns	-125.1 to 80.26	***	-129.3 to -39.32
**C9 vs C17**	***	44.33 to 98.14	ns	-6.531 to 14.12	ns	-69.31 to 185.3	ns	-109.4 to 2.229
**C9 vs C18**	***	-56.50 to -10.51	ns	-4.992 to 12.66	ns	-95.47 to 122.3	ns	-50.32 to 45.11
**C10 vs C11**	***	15.94 to 62.48	ns	-10.88 to 6.978	ns	-69.84 to 150.2	***	57.22 to 153.8
**C10 vs C12**	**	-52.12 to -3.919	ns	-11.58 to 6.917	ns	-224.2 to 3.672	***	36.47 to 136.5
**C10 vs C13**	***	50.73 to 101.3	ns	-15.39 to 0.05947	ns	-59.93 to 130.6	***	49.17 to 132.7
**C10 vs C14**	***	40.48 to 83.71	**	-18.33 to -1.738	*	1.790 to 207.0	***	55.54 to 145.2
**C10 vs C15**	ns	-1.414 to 40.39	***	-19.16 to -3.114	ns	-116.3 to 82.40	***	40.02 to 126.8
**C10 vs C16**	***	26.33 to 66.59	ns	-14.88 to 4.516	ns	-128.6 to 110.3	ns	-15.03 to 89.84
**C10 vs C17**	***	39.14 to 98.88	ns	-20.37 to 2.560	ns	-69.96 to 212.4	*	6.160 to 130.1
**C10 vs C18**	***	-62.13 to -9.323	ns	-19.00 to 1.271	ns	-98.19 to 151.5	***	64.34 to 173.9
**C11 vs C12**	***	-90.44 to -44.02	ns	-9.287 to 8.530	***	-260.2 to -40.69	ns	-67.20 to 29.13
**C11 vs C13**	***	12.36 to 61.22	ns	-13.03 to 1.601	ns	-95.03 to 85.38	ns	-54.11 to 24.99
**C11 vs C14**	*	2.263 to 43.50	*	-15.99 to -0.1661	ns	-33.69 to 162.2	ns	-47.90 to 37.66
**C11 vs C15**	ns	-39.60 to 0.1515	**	-16.81 to -1.556	ns	-151.7 to 37.39	ns	-63.35 to 19.12
**C11 vs C16**	ns	-11.81 to 26.31	ns	-12.60 to 6.144	ns	-164.8 to 66.13	***	-118.8 to -17.41
**C11 vs C17**	*	0.6396 to 58.95	ns	-18.14 to 4.240	ns	-106.8 to 168.9	ns	-97.87 to 23.13
**C11 vs C18**	***	-100.5 to -49.34	ns	-16.73 to 2.914	ns	-134.5 to 107.5	ns	-39.49 to 66.73
**C12 vs C13**	***	78.80 to 129.2	ns	-13.04 to 2.366	***	50.65 to 240.5	ns	-37.16 to 46.10
**C12 vs C14**	***	68.56 to 111.7	ns	-15.97 to 0.5704	***	112.3 to 317.0	ns	-30.81 to 58.63
**C12 vs C15**	***	26.67 to 68.35	*	-16.80 to -0.8065	ns	-5.780 to 192.3	ns	-46.32 to 40.16
**C12 vs C16**	***	54.41 to 94.54	ns	-12.53 to 6.827	ns	-18.13 to 220.3	ns	-101.4 to 3.260
**C12 vs C17**	***	67.20 to 126.9	ns	-18.02 to 4.875	***	40.52 to 322.5	ns	-80.22 to 43.54
**C12 vs C18**	ns	-34.06 to 18.64	ns	-16.64 to 3.583	*	12.31 to 261.5	ns	-22.03 to 87.33
**C13 vs C14**	ns	-36.76 to 8.946	ns	-8.889 to 4.157	ns	-11.96 to 150.1	ns	-25.83 to 44.71
**C13 vs C15**	***	-78.69 to -34.33	ns	-9.643 to 2.704	ns	-129.2 to 24.56	ns	-40.93 to 25.82
**C13 vs C16**	***	-51.00 to -8.084	ns	-5.751 to 10.72	ns	-146.0 to 57.01	**	-98.06 to -9.016
**C13 vs C17**	ns	-37.77 to 23.78	ns	-11.49 to 9.018	ns	-90.48 to 162.3	ns	-78.25 to 32.63
**C13 vs C18**	***	-139.2 to -84.30	ns	-9.939 to 7.547	ns	-116.5 to 99.07	ns	-19.09 to 75.45
**C14 vs C15**	***	-60.51 to -24.70	ns	-7.975 to 5.768	***	-207.2 to -35.56	ns	-54.15 to 20.16
**C14 vs C16**	ns	-32.63 to 1.364	ns	-3.921 to 13.62	*	-222.0 to -5.129	***	-110.4 to -15.56
**C14 vs C17**	ns	-20.94 to 34.77	ns	-9.559 to 11.82	ns	-165.2 to 98.84	ns	-90.04 to 25.54
**C14 vs C18**	***	-121.9 to -73.72	ns	-8.080 to 10.42	ns	-192.1 to 36.55	ns	-31.27 to 68.75
**C15 vs C16**	***	10.89 to 43.06	ns	-2.560 to 14.47	ns	-97.59 to 113.2	ns	-92.01 to 0.04495
**C15 vs C17**	***	22.21 to 76.82	ns	-8.246 to 12.71	ns	-41.29 to 217.7	ns	-71.91 to 41.40
**C15 vs C18**	***	-78.68 to -31.75	ns	-6.733 to 11.28	ns	-67.81 to 155.0	ns	-12.96 to 84.42
**C16 vs C17**	ns	-4.171 to 49.27	ns	-15.53 to 8.092	ns	-65.08 to 225.9	ns	-33.13 to 94.59
**C16 vs C18**	***	-105.0 to -59.41	ns	-14.21 to 6.847	ns	-93.85 to 165.5	***	24.81 to 138.6
**C17 vs C18**	***	-136.5 to -73.02	ns	-12.13 to 12.21	ns	-194.5 to 105.3	ns	-14.81 to 116.8

Effects of nicotine, radial extracorporeal shock waves and recovery time were analyzed using one-way ANOVA followed by Bonferroni's multiple comparison test. *$p < 0.05$; **$p < 0.01$; ***$p < 0.001$; ns, not significant.

Table S5. Summary of absolute mean values, % of control, and corresponding 95% confidence intervals (CIs) for absolute peristaltic speed, wavelength, omega bends, and reversal frequency in Assay D.

		D1	D2	D3	D4	D5	D6
Absolute peristaltic speed	µm/s	126.3 ± 3.38 (257)	128 ± 4.51 (168)	137.6 ± 4.54 (145)	61.75 ± 4.16 (249)	31.12 ± 2.32 (413)	27.42 ± 1.67 (402)
	% of control	100.00 ± 2.68 (257)	101.40 ± 3.57(168)	109.0 ± 3.60 (145)	48.89 ± 3.29 (249)	24.64 ± 1.84 (413)	21.71 ± 1.32 (402)
	95 % CI (% of control)	94.7 – 105.3	94.3 – 108.4	101.9 – 116.1	42.4 – 55.4	21.0 – 28.3	19.1 – 24.3
Wavelength	µm	351.10 ± 4.26 (257)	345.60 ± 4.66 (168)	335.40 ± 4.41 (145)	383.40 ± 7.82 (249)	384.40 ± 5.64 (413)	407.70 ± 5.82 (402)
	% of control	100.00 ± 1.21 (257)	98.42 ± 1.33 (168)	95.53 ± 1.26 (145)	109.20 ± 2.23 (249)	109.50 ± 1.61 (413)	116.10 ± 1.66 (402)
	95 % CI (% of control)	97.6 – 102.4	95.8 – 101.0	93.1 – 98.0	104.8 – 113.6	106.3 – 112.7	112.9 – 119.4
Omega bends	average/frame	0.1090 ± 0.0090 (245)	0.0798 ± 0.0097 (168)	0.1342 ± 0.0126 (139)	0.0346 ± 0.0070 (249)	0.0362 ± 0.0065 (406)	0.0285 ± 0.0059 (398)
	% of control	100.00 ± 8.25 (245)	73.44 ± 8.87 (168)	123.30 ± 11.61 (139)	31.89 ± 6.46 (249)	33.30 ± 5.93 (406)	26.19 ± 5.39 (398)
	95 % CI (% of control)	83.8 – 116.2	55.9 – 91.0	100.3 – 146.3	19.2 – 44.6	21.6 – 45.0	15.6 – 36.8
Reversals	average/frame	0.0082 ± 0.0005 (257)	0.0080 ± 0.0006 (168)	0.0094 ± 0.0007 (145)	0.0146 ± 0.0005 (249)	0.0173 ± 0.0003 (413)	0.0169 ± 0.0003 (402)
	% of control	100.50 ± 4.88 (257)	95.53 ± 5.87 (168)	106.20 ± 7.39 (145)	163.60 ± 4.71 (249)	193.70 ± 3.12 (413)	186.30 ± 3.29 (402)
	95 % CI (% of control)	90.87 - 110.1	83.95 - 107.1	91.57 - 120.8	154.3 - 172.8	187.5 - 199.8	179.8 - 192.8

Data are presented as mean ± SEM (n) for absolute values and % of control.

Table S6 Summary of absolute mean values, % of control, and corresponding 95% confidence intervals (CIs) for absolute peristaltic speed, wavelength, omega bends, and reversal frequency in Assay E.

		E1	E2	E3	E4	E5	E6
Absolute peristaltic speed	µm/s	83.74 ± 3.94 (131)	103.4 ± 6.15 (90)	96.87 ± 3.67 (179)	63.83 ± 7.77 (65)	49.79 ± 4.22 (99)	10.11 ± 1.62 (136)
	% of control	100.00 ± 4.71 (131)	123.50 ± 7.34 (90)	115.7 ± 4.38 (179)	77.12 ± 9.18 (65)	59.47 ± 5.04 (99)	12.07 ± 1.93 (136)
	95 % CI (% of control)	90.7 – 109.3	108.9 – 138.1	107.0 – 124.3	58.8 – 95.5	49.5 – 69.5	8.2 – 15.9
Wavelength	µm	323.80 ± 3.93 (131)	332.30 ± 4.27 (90)	316.60 ± 2.44 (179)	334.40 ± 8.64 (65)	345.90 ± 6.75 (99)	384.10 ± 9.21 (136)
	% of control	100.00 ± 1.21 (131)	102.60 ± 1.32 (90)	97.78 ± 0.75 (179)	103.30 ± 2.67 (65)	106.80 ± 2.08 (99)	118.60 ± 2.84 (136)
	95 % CI (% of control)	97.6 – 102.4	100.0 – 105.2	96.3 – 99.3	97.9 – 108.6	102.7 – 111.0	113.0 – 124.3
Omega bends	average/frame	0.0715 ± 0.0086 (131)	0.0671 ± 0.0111 (90)	0.0579 ± 0.0068 (179)	0.0658 ± 0.0147 (65)	0.0419 ± 0.0091 (99)	0.0193 ± 0.0091 (136)
	% of control	100.00 ± 12.04 (131)	93.76 ± 15.50 (90)	81.00 ± 9.51 (179)	79.79 ± 16.82 (65)	58.59 ± 12.70 (99)	27.01 ± 12.72 (136)
	95 % CI (% of control)	76.2 – 123.8	63.0 – 124.6	62.2 – 99.8	46.2 – 113.4	33.4 – 83.8	1.9 – 52.2
Reversals	average/frame	0.0070 ± 0.0007 (131)	0.0073 ± 0.0008 (90)	0.0060 ± 0.0005 (179)	0.0138 ± 0.0010 (65)	0.0111 ± 0.0009 (99)	0.0171 ± 0.0005 (136)
	% of control	99.95 ± 9.26 (131)	103.10 ± 11.81 (90)	84.76 ± 7.08 (179)	196.00 ± 13.96 (65)	156.70 ± 12.09 (99)	242.00 ± 6.70 (136)
	95 % CI (% of control)	81.63 - 118.3	79.63 -126.6	70.78 - 98.74	168.1 -223.8	132.7 - 180.7	228.8 - 255.3

Data are presented as mean ± SEM (n) for absolute values and % of control.

Table S7. Details of the statistical analysis for Assays D and E, including p values and 95% confidence intervals of differences (CIs of diff) for peristaltic speed, wavelength, omega bends, and reversal frequency.

	Absolut peristaltic speed		Wavelength		Omega bends		Reversals	
P value (ANOVA):	< 0.0001		< 0.0001		< 0.0001		< 0.0001	
	Bonferroni's Multiple Comparison Test							
	P value	95% CI of diff	P value	95% CI of diff	P value	95% CI of diff	P value	95% CI of diff
**D1 vs D2**	ns	-16,43 to 13,70	ns	-7,326 to 10,49	ns	-15,04 to 68,16	ns	-22,53 to 32,42
**D1 vs D3**	ns	-24,73 to 6,816	ns	-4,854 to 13,79	ns	-67,40 to 20,81	ns	-34,45 to 23,07
**D1 vs D4**	***	37,60 to 64,62	**	-17,18 to -1,211	***	30,74 to 105,5	***	-87,72 to -38,47
**D1 vs D5**	***	63,29 to 87,43	***	-16,63 to -2,361	***	33,10 to 100,3	***	-115,2 to -71,19
**D1 vs D6**	***	66,16 to 90,42	***	-23,29 to -8,947	***	40,08 to 107,5	***	-108,0 to -63,72
**D1 vs E1**	ns	-16,30 to 16,30	ns	-9,637 to 9,637	ns	-44,95 to 44,96	ns	-29,20 to 30,26
**D1 vs E2**	**	-42,10 to -4,892	ns	-13,62 to 8,371	ns	-44,96 to 57,43	ns	-36,54 to 31,30
**D1 vs E3**	*	-30,47 to -0,8987	ns	-6,515 to 10,96	ns	-21,83 to 59,84	ns	-11,25 to 42,67

**D1 vs E4**	*	1,791 to 43,96	ns	-15,74 to 9,189	ns	-38,09 to 78,52	***	-133,9 to -57,03
**D1 vs E5**	***	22,57 to 58,50	ns	-17,45 to 3,789	ns	-8,053 to 90,87	***	-89,02 to -23,50
**D1 vs E6**	***	71,83 to 104,0	***	-28,15 to -9,109	***	28,58 to 117,4	***	-170,9 to -112,2
**D2 vs D3**	ns	-24,81 to 9,622	ns	-7,286 to 13,07	*	-97,48 to -2,236	ns	-42,03 to 20,76
**D2 vs D4**	***	37,31 to 67,64	**	-19,74 to -1,811	*	0,08747 to 83,02	***	-95,69 to -40,39
**D2 vs D5**	***	62,83 to 90,62	***	-19,29 to -2,859	*	2,043 to 78,25	***	-123,5 to -72,80
**D2 vs D6**	***	65,70 to 93,61	***	-25,94 to -9,450	**	9,039 to 85,46	***	-116,2 to -65,34
**D2 vs E1**	ns	-16,34 to 19,07	ns	-12,04 to 8,883	ns	-74,96 to 21,86	ns	-36,69 to 27,87
**D2 vs E2**	*	-41,97 to -2,290	ns	-15,93 to 7,522	ns	-74,58 to 33,93	ns	-43,74 to 28,61
**D2 vs E3**	ns	-30,63 to 1,995	ns	-8,999 to 10,29	ns	-52,17 to 37,06	ns	-18,98 to 40,52
**D2 vs E4**	*	2,057 to 46,43	ns	-17,97 to 8,258	ns	-67,36 to 54,66	***	-140,9 to -59,97
**D2 vs E5**	***	22,66 to 61,14	ns	-19,78 to 2,964	ns	-37,77 to 67,47	***	-96,29 to -26,11
**D2 vs E6**	***	71,78 to 106,8	***	-30,56 to -9,853	ns	-1,477 to 94,34	***	-178,5 to -114,6
**D3 vs D4**	***	44,20 to 75,93	***	-23,04 to -4,285	***	47,44 to 135,4	***	-86,33 to -28,47
**D3 vs D5**	***	69,66 to 98,98	***	-22,63 to -5,298	***	49,19 to 130,8	***	-114,2 to -60,77
**D3 vs D6**	***	72,54 to 102,0	***	-29,28 to -11,89	***	56,19 to 138,0	***	-107,0 to -53,32
**D3 vs E1**	ns	-9,349 to 27,27	ns	-15,29 to 6,351	ns	-27,27 to 73,88	ns	-27,16 to 39,60
**D3 vs E2**	ns	-34,92 to 5,845	ns	-19,14 to 4,952	ns	-26,66 to 85,73	ns	-34,09 to 40,23
**D3 vs E3**	ns	-23,69 to 10,24	ns	-12,28 to 7,784	ns	-4,652 to 89,26	ns	-9,540 to 52,34
**D3 vs E4**	***	9,165 to 54,51	ns	-21,14 to 5,655	ns	-19,23 to 106,3	***	-131,1 to -48,45
**D3 vs E5**	***	29,69 to 69,29	ns	-23,00 to 0,4038	**	10,09 to 119,3	***	-86,67 to -14,46
**D3 vs E6**	***	78,76 to 115,0	***	-33,81 to -12,38	***	46,19 to 146,4	***	-168,9 to -102,8
**D4 vs D5**	***	12,07 to 36,44	ns	-7,503 to 6,902	ns	-34,84 to 32,02	***	-52,32 to -7,882
**D4 vs D6**	***	14,93 to 39,43	ns	-14,16 to 0,3154	ns	-27,86 to 39,26	*	-45,07 to -0,4063
**D4 vs E1**	***	-67,50 to -34,72	ns	-0,4961 to 18,88	***	-112,9 to -23,28	***	33,73 to 93,52
**D4 vs E2**	***	-93,28 to -55,92	ns	-4,472 to 17,61	**	-113,0 to -10,79	***	26,41 to 94,53
**D4 vs E3**	***	-81,68 to -51,91	***	2,621 to 20,21	**	-89,81 to -8,410	***	51,67 to 105,9
**D4 vs E4**	***	-49,39 to -7,078	ns	-6,585 to 18,42	ns	-106,1 to 10,30	ns	-70,96 to 6,193
**D4 vs E5**	ns	-28,62 to 7,470	ns	-8,303 to 13,03	ns	-76,05 to 22,64	ns	-26,07 to 39,74
**D4 vs E6**	***	20,63 to 53,02	ns	-19,01 to 0,1369	ns	-39,41 to 49,16	***	-108,0 to -48,95
**D5 vs D6**	ns	-7,712 to 13,57	*	-12,91 to -0,3342	ns	-22,19 to 36,40	ns	-12,04 to 26,77
**D5 vs E1**	***	-90,59 to -60,13	*	0,4917 to 18,49	***	-108,4 to -24,96	***	65,96 to 121,5
**D5 vs E2**	***	-116,5 to -81,19	ns	-3,574 to 17,31	**	-108,9 to -12,08	***	58,36 to 122,8
**D5 vs E3**	***	-104,6 to -77,45	***	3,684 to 19,75	**	-84,96 to -10,44	***	84,13 to 133,7
**D5 vs E4**	***	-72,75 to -32,22	ns	-5,760 to 18,20	ns	-102,4 to 9,367	ns	-39,23 to 34,68
**D5 vs E5**	***	-51,82 to -17,83	ns	-7,382 to 12,71	ns	-71,85 to 21,26	**	5,946 to 67,93
**D5 vs E6**	ns	-2,445 to 27,59	*	-18,01 to -0,2596	ns	-34,86 to 47,44	***	-75,75 to -20,99
**D6 vs E1**	***	-93,57 to -63,01	***	7,086 to 25,15	***	-115,6 to -31,97	***	58,50 to 114,2
**D6 vs E2**	***	-119,5 to -84,07	***	3,024 to 23,96	***	-116,1 to -19,10	***	50,92 to 115,5
**D6 vs E3**	***	-107,6 to -80,33	***	10,28 to 26,41	***	-92,18 to -17,43	***	76,66 to 126,4
**D6 vs E4**	***	-75,72 to -35,11	*	0,8418 to 24,84	ns	-109,5 to 2,336	ns	-46,66 to 27,38
**D6 vs E5**	***	-54,80 to -20,72	ns	-0,7848 to 19,36	ns	-79,05 to 14,24	ns	-1,498 to 60,65
**D6 vs E6**	ns	-5,425 to 24,71	ns	-11,42 to 6,394	ns	-42,07 to 40,43	***	-83,21 to -28,26
**E1 vs E2**	**	-44,29 to -2,701	ns	-14,91 to 9,666	ns	-50,63 to 63,10	ns	-41,07 to 34,76
**E1 vs E3**	ns	-33,15 to 1,778	ns	-8,097 to 12,55	ns	-28,76 to 66,75	ns	-16,66 to 47,02
**E1 vs E4**	ns	-0,1653 to 45,92	ns	-16,89 to 10,35	ns	-43,14 to 83,55	***	-138,0 to -53,99
**E1 vs E5**	***	20,31 to 60,76	ns	-18,78 to 5,125	ns	-13,91 to 96,71	***	-93,67 to -19,91
**E1 vs E6**	***	69,34 to 106,5	***	-29,62 to -7,638	***	22,14 to 123,8	***	-176,0 to -108,2
**E2 vs E3**	ns	-11,82 to 27,44	ns	-6,751 to 16,45	ns	-40,90 to 66,44	ns	-17,45 to 54,12
**E2 vs E4**	***	21,65 to 71,09	ns	-15,26 to 13,96	ns	-53,94 to 81,89	***	-137,9 to -47,77
**E2 vs E5**	***	41,91 to 86,15	ns	-17,28 to 8,869	ns	-25,32 to 95,66	***	-93,97 to -13,30
**E2 vs E6**	***	90,79 to 132,1	***	-28,20 to -3,805	**	10,32 to 123,2	***	-176,6 to -101,3

**E3 vs E4**	***	16,57 to 60,56	ns	-18,50 to 7,501	ns	-59,28 to 61,70	***	-151,3 to -71,08
**E3 vs E5**	***	37,20 to 75,24	ns	-20,30 to 2,189	ns	-29,62 to 74,43	***	-106,7 to -37,28
**E3 vs E6**	***	86,34 to 120,9	***	-31,06 to -10,64	**	6,741 to 101,2	***	-188,8 to -125,8
**E4 vs E5**	ns	-6,589 to 41,90	ns	-17,89 to 10,78	ns	-45,42 to 87,81	ns	-4,996 to 83,43
**E4 vs E6**	***	42,15 to 87,96	**	-28,89 to -1,817	ns	-10,18 to 115,7	*	-87,85 to -4,334
**E5 vs E6**	***	27,33 to 67,46	ns	-23,66 to 0,06171	ns	-23,29 to 86,45	***	-121,9 to -48,72

Effects of carbachol and radial extracorporeal shock waves were analyzed using one-way ANOVA followed by Bonferroni's multiple comparison test. *$p < 0.05$; **$p < 0.01$; ***$p < 0.001$; ns, not significant.
